# Validity and Reliability of the Arabic Version of the Patient-Specific Functional Scale in Individuals with Lateral Elbow Pain: A Cross-Sectional Study

**DOI:** 10.3390/healthcare14182997

**Published:** 2026-09-14

**Authors:** Shareef Khawaji, Hadeel R. Bakhsh, Abdullah Alzahrani, Maryam Farahat, Fatmah Hasani

**Affiliations:** 1 Security Forces Hospital Program, General Directorate of Medical Services, Ministry of Interior, Riyadh 11481, Saudi Arabiafatmahhasani@gmail.com (F.H.); 2 Department of Rehabilitation Sciences, College of Health and Rehabilitation Sciences, Princess Nourah bint Abdulrahman University, Riyadh 11671, Saudi Arabia

**Keywords:** lateral elbow pain, Patient-Specific Functional Scale, psychometric properties, reliability, construct validity, Arabic version, patient-reported outcome measures

## Abstract

**Background/objectives**: Lateral elbow pain (LEP) is a common musculoskeletal disorder that can substantially affect upper limb function and daily activities. Patient-reported outcome measures (PROMs) are essential for evaluating functional limitations from the patient’s perspective. The Patient-Specific Functional Scale (PSFS) is a patient-centered instrument designed to capture individualized activity limitations. The objective is to evaluate the construct validity, test–retest reliability, and measurement error of the Arabic version of the Patient-Specific Functional Scale (PSFS-Ar) in Arabic-speaking individuals with lateral elbow pain. **Methods**: A cross-sectional study was conducted in the physiotherapy department at Security Forces Hospital in Riyadh, Saudi Arabia. Sixty-one adults diagnosed with lateral elbow pain completed the PSFS-Ar, the Numeric Pain Rating Scale (NPRS-Ar), and the Upper Extremity Functional Index (UEFI-Ar). Construct validity was examined using Spearman’s correlation coefficients and hypothesis testing. Dependent correlations were compared using Steiger’s Z test. Test–retest reliability was evaluated in a subgroup of 30 participants using the intraclass correlation coefficient (ICC2,1). Measurement error was quantified using the standard error of measurement (SEM) and minimal detectable change at the 95% confidence level (MDC95). Floor and ceiling effects were also assessed. **Results**: The PSFS-Ar demonstrated a moderate point estimate of test–retest reliability (ICC = 0.65, 95% CI: 0.35–0.82). The SEM was 1.03 points, and the MDC95 was 2.86 points. Neither of the two prespecified construct-validity hypotheses was confirmed; therefore, construct validity was not supported according to the prespecified hypothesis-testing criterion. The PSFS-Ar showed a weak positive correlation with the UEFI-Ar (r_s_ = 0.35, *p* = 0.005) and a negligible, nonsignificant correlation with the NPRS-Ar (r_s_ = −0.17, *p* = 0.200). No floor or ceiling effects were observed. **Conclusions**: The PSFS-Ar demonstrated a moderate but imprecise test–retest reliability estimate, while construct validity was not supported according to the prespecified hypothesis-testing criterion. The wide ICC confidence interval, small reliability sample, systematic change between assessments, variable retest interval, and absence of a validated stability anchor limit confidence in these findings. Further studies using larger samples and standardized retest procedures are required.

## 1. Introduction

Lateral elbow pain (LEP), commonly referred to as lateral epicondylalgia, is a common musculoskeletal disorder primarily affecting the lateral epicondyle of the humerus. In more persistent presentations, symptoms may extend proximally or distally, and the clinical presentation may involve contributors beyond local tendon pathology; however, symptom distribution is multifactorial, and kinetic-chain or neural involvement should not be assumed in every individual [1]. Clinically, LEP is characterized by localized tenderness over the lateral epicondyle. Pain is typically reproduced by exerting pressure on the lateral epicondyle, resisting wrist and/or third finger extension and stretching of the wrist extensors [1].

The LEP encompasses a spectrum of degenerative and inflammatory disorders affecting the joints, peripheral nerves, tendons, ligaments, muscles, and supporting vascular structures [1]. It most commonly affects individuals between 35 and 54 years of age, with an estimated prevalence of 1% to 3% in the general population, with higher rates reported among occupational groups performing repetitive upper-limb loading [1,2]. Rehabilitation for LEP emphasizes a biopsychosocial and patient-centered framework integrating load management, progressive exercise therapy, education, and functional restoration. Collaborative goal setting is central to optimizing adherence and clinical outcomes, consistent with contemporary practice guidelines (CPGs) for managing musculoskeletal conditions [3,4].

Given the multidimensional nature of LEP and its impact on pain, function, and quality of life, comprehensive outcome evaluation is essential. Patient-reported outcome measures (PROMs) are therefore necessary for evaluating the effectiveness of the rehabilitation interventions. The PROMs capture patients’ subjective experiences of pain, function, and health-related quality of life, thereby assisting in measuring functional improvements from the patient’s perspective. Their use helps ensure that the care provided is meaningful, and significant to those receiving it [5,6], yet clinicians often face challenges in selecting outcome measures that accurately capture patient-centered functional limitations [7].

However, many of these measures rely on predefined items that may not fully capture the specific functional difficulties experienced by individual patients, particularly those engaged in diverse occupational, sporting, or high-demand activities. Therefore, individualized outcome measures such as the Patient-Specific Functional Scale (PSFS) [8] may offer additional clinical value by enabling patients to identify and rate activities that are personally meaningful and directly affected by their condition.

The PSFS is widely used in both clinical practice and research within the context of rehabilitation [8]. The PSFS is considered a simple, efficient, relatively quick and patient-centered outcome measure. It focuses on activities that are important to the patient rather than those predefined by healthcare professionals [9]. Patients generate items and rate activities they find difficult, allowing them to report the degree of functional limitation at baseline and during follow-up sessions. This approach enhances clinical relevance and sensitivity to meaningful change [3,10]. According to Clinical Practice Guidelines for Lateral Elbow Pain and Muscle Functions Impairment CPG, individuals engaged in high-demand activities may particularly benefit from the PSFS [11].

The PSFS has demonstrated strong intrinsic psychometric properties across various musculoskeletal conditions, including high responsiveness, good test–retest reliability, acceptable measurement error, and evidence supporting its construct validity [8]. With an emphasis on upper extremity disorders, the PSFS has been identified as a reliable measure of functional limitation such as difficulty lifting, reaching, or grasping [2]. Supported by high-quality evidence and adequate measurement properties, the PSFS can be regarded as a reliable, responsive, and valid tool for evaluating functional change in patients with upper extremity disorders [5]. Furthermore, its cross-cultural adaptation into the Arabic language (PSFS-Ar) has been reported as both comprehensible and clinically robust, demonstrating excellent test–retest reliability and construct validity when applied to individuals with musculoskeletal and neurological disorders [3].

Although the PSFS-Ar has been evaluated in other clinical populations, its measurement properties have not been specifically established in Arabic-speaking individuals with lateral elbow pain. Given the condition-specific and individualized nature of functional limitations, further evaluation in this population is warranted to support accurate score interpretation [12,13]. Therefore, the present study aimed to evaluate the construct validity, test–retest reliability, and measurement error of the PSFS-Ar in individuals with lateral elbow pain in Saudi Arabia. Applying the PSFS-Ar to the specific functional challenges associated with lateral elbow pain represents a logical progression of this work [14].

## 2. Materials and Methods

### 2.1. Study Design and Setting

This study employed a cross-sectional design to evaluate the psychometric properties of the PSFS-Ar in individuals with LEP. Participants were recruited from the physiotherapy department at the Security Forces Hospital Program, a governmental tertiary care facility in Riyadh, Saudi Arabia, between June and December 2025.

The sample size was determined during the study planning based on recommendations from the Consensus-based Standards for the Selection of Health Measurement Instruments [15]. A minimum of 50 participants is considered adequate for the evaluation of construct validity and floor/ceiling effects, while at least 30 participants are recommended for test–retest reliability analysis. Accordingly, a total sample of 61 participants was recruited to ensure sufficient power for the construct validity analyses, with a subsample of 30 participants completing the retest assessment.

### 2.2. Participants

A consecutive sampling strategy was used to minimize selection bias, whereby all eligible patients presenting during the study period were invited to participate. The study was conducted in accordance with the Declaration of Helsinki and received ethical approval from the Institutional Review Board (IRB) of the Security Forces Hospital Program (Registration No. H-01-R-069).

Eligible participants were adults aged ≥18 years with a clinical diagnosis of lateral elbow pain. The diagnosis was established by the referring family medicine or orthopedic clinician and confirmed during physiotherapy assessment by tenderness over the lateral epicondyle and reproduction of familiar lateral elbow pain during resisted wrist extension and/or resisted middle-finger extension. Participants were excluded when the assessment suggested cervical radiculopathy, a recent upper-limb fracture, or another condition more likely to explain their symptoms. All participants provided written informed consent prior to data collection.

### 2.3. Outcome Measures

Three validated Arabic-language patient-reported outcome measures were administered to assess pain and function. Sociodemographic and clinical characteristics were also collected, including age, sex, employment status, hand dominance, affected side, and symptom duration.

#### 2.3.1. Patient-Specific Functional Scale (PSFS-Ar)

The PSFS-Ar is a patient-centered outcome measure that assesses activity limitations specific to the individual. Participants were asked to identify up to five activities that they were unable to perform or had difficulty performing due to their condition. Each activity was rated on an 11-point numeric scale ranging from 0 (“unable to perform”) to 10 (“able to perform at pre-injury level”). The total PSFS-Ar score was calculated as the mean of the activity scores, with higher scores indicating better functional performance. Consistent with the individualized nature of the PSFS, item content varies between participants. At T2, participants rated the same patient-nominated activities identified at T1. Although participants could report newly developed problematic activities, these were not included in the test–retest reliability analysis, which was based exclusively on ratings of the original T1 activities [8]. The PSFS-Ar has previously demonstrated good reliability and construct validity in Arabic-speaking populations with musculoskeletal and neurological conditions [3].

#### 2.3.2. Numeric Pain Rating Scale (NPRS-Ar)

Pain intensity was assessed using the Arabic version of the Numeric Pain Rating Scale (NPRS-Ar). Participants rated their average pain intensity over the past 24 h on an 11-point scale ranging from 0 (“no pain”) to 10 (“worst imaginable pain”). The NPRS-Ar is a widely used measure with established validity and reliability in Arabic-speaking populations [16].

#### 2.3.3. Upper Extremity Functional Index (UEFI-Ar)

Upper limb function was assessed using the Arabic version of the Upper Extremity Functional Index (UEFI-Ar). This questionnaire consists of 20 items, each rated on a 5-point Likert scale (0 = extreme difficulty or inability to perform, 4 = no difficulty). Total scores range from 0 to 80, with higher scores indicating better upper extremity function. The UEFI-Ar has demonstrated excellent internal consistency, test–retest reliability, and construct validity, making it an appropriate comparator for evaluating the construct validity of the PSFS-Ar [17].

### 2.4. Procedure

Eligible participants were identified through the physiotherapy department at the Security Forces Hospital Program during routine clinical care. Treating physiotherapists initially screened patients for eligibility based on the inclusion and exclusion criteria. Patients who met the criteria were then approached by a member of the research team, who provided detailed information about the study and obtained written informed consent.

Data collection was conducted by three trained physiotherapists who were part of the research team and had a minimum of four years of clinical experience. To ensure standardization and consistency in data collection, all assessors participated in a structured training session prior to study initiation. This session covered study procedures, administration of outcome measures, and guidelines for responding to participant queries.

At baseline (T1), participants completed the PSFS-Ar, NPRS-Ar, and UEFI-Ar in a supervised setting to ensure completeness and accuracy of responses. Standardized instructions were provided, and clarification was offered when necessary, without influencing participants’ responses.

For the test–retest reliability assessment, a consecutive subsample of 30 participants was recruited from the 61 participants who completed the baseline assessment. Participants were included consecutively in the reliability subsample according to their order of recruitment until the planned sample of 30 was reached. These participants completed the PSFS-Ar at T2, 5–14 days after the baseline assessment. The remaining 31 participants were not included in the retest assessment.

At T2, participants rated the same patient-nominated activities identified at T1 and were asked whether they had experienced a substantial change in their elbow condition since T1. Participants reporting substantial change were intended to be excluded. However, no validated stability anchor or predefined quantitative threshold was used, and routine clinical care was not standardized during the retest interval. Therefore, subtle clinical change between T1 and T2 could not be excluded.

Two post hoc sensitivity analyses were conducted to explore whether the primary reliability estimate was sensitive to progressively shorter retest intervals. The ICC(2,1) was recalculated for participants retested within <11 days and <9 days. These thresholds were not prespecified and were not based on established clinical cutoffs; they were selected solely as exploratory restrictions of the observed retest interval. Accordingly, these analyses were considered sensitivity analyses only and were not used to redefine the primary reliability estimate.

All questionnaires were completed in paper format and subsequently entered into a secure electronic database. Data entry accuracy was ensured through double-checking and random verification of records. Participant confidentiality was maintained by assigning anonymized identification codes, and all data were stored in accordance with institutional data protection policies. Treatment provided during the study period followed routine clinical practice and was not standardized or influenced by the study protocol.

### 2.5. Data Analysis

All data were entered into Microsoft Excel and subsequently analyzed using IBM SPSS Statistics (Version 29.0; IBM Corp., Armonk, NY, USA). Data accuracy was ensured through visual inspection and random verification of entries. Statistical significance was set at *p* < 0.05 (two-tailed), and 95% confidence intervals (CIs) were reported where appropriate.

#### 2.5.1. Descriptive Analysis

Descriptive statistics were used to summarize participants’ sociodemographic and clinical characteristics, as well as outcome measure scores. Categorical variables were presented as frequencies and percentages. Continuous variables were assessed for normality using visual inspection (histograms and Q–Q plots) and appropriate normality tests. Normally distributed data were reported as mean ± standard deviation (SD), whereas non-normally distributed data were reported as median and interquartile range (IQR).

#### 2.5.2. Psychometric Assessment

Construct Validity—The PSFS-Ar was evaluated using hypothesis testing, in accordance with COSMIN recommendations [15]. A priori hypotheses were formulated regarding the expected direction and magnitude of correlations between the PSFS-Ar and comparator instruments. Specifically, it was hypothesized that:

- PSFS-Ar scores would demonstrate a moderate positive correlation with the UEFI-Ar (functional status), and
- a weak to moderate negative correlation with the NPRS-Ar (pain intensity).

Given that at least one of the variables did not meet parametric assumptions, Spearman’s rank correlation coefficient (rs) was used. Correlation strength was interpreted as follows: <0.20 = negligible, 0.20–0.39 = weak, 0.40–0.59 = moderate, and ≥0.60 = strong. As a supplementary analysis, Steiger’s Z test was used to compare the two dependent correlations (PSFS-Ar–UEFI-Ar and PSFS-Ar–NPRS-Ar) to examine whether the PSFS-Ar was more strongly associated with functional status than with pain intensity. This analysis was considered supplementary and was not used to determine whether the prespecified construct-validity hypotheses were confirmed [18]. Construct validity was considered supported when at least 75% of the predefined hypotheses were confirmed, in accordance with COSMIN criteria [19].

Test–retest reliability—The test–retest reliability of the PSFS-Ar was assessed in participants who completed the instrument at two time points (T1 and T2) using the Intraclass Correlation Coefficient (ICC) based on a two-way random-effects model with absolute agreement [ICC(2,1)] [20,21]. The ICC (2,1) model was selected because each assessment produced a single score intended for independent clinical interpretation, and absolute agreement was required to evaluate consistency between repeated measurements. ICC values were interpreted as follows: <0.50 = poor reliability, 0.50–0.75 = moderate reliability, 0.75–0.90 = good reliability, ≥0.90 = excellent reliability [22].

Measurement error—Measurement error was quantified using the Standard Error of Measurement (SEM) and the Minimal Detectable Change at the 95% confidence level (MDC_95_). The SEM reflects the absolute precision of individual scores. The SEM was calculated using the baseline standard deviation of PSFS-Ar scores in the reliability subgroup (SD = 1.72): SEM = SD baseline × √(1 − ICC) [23]. The MDC_95_ represents the smallest change that can be interpreted as a true change beyond measurement error and was calculated as: MDC_95_ = SEM × 1.96 × √2 [24].

Floor and ceiling—Floor and ceiling were assessed by calculating the proportion of participants achieving the lowest and highest possible PSFS-Ar scores, respectively. Effects were considered present if more than 15% of participants achieved either extreme score. The absence of such effects indicates adequate score distribution and supports the instrument’s ability to detect variability across different levels of function [25].

Exploratory subgroup comparisons were conducted to describe possible variation in PSFS-Ar scores according to sex, employment status, symptom duration, and affected side. These analyses were not based on prespecified hypotheses, and no adjustment for multiple comparisons was applied; therefore, the findings were considered hypothesis-generating rather than confirmatory.

## 3. Results

### 3.1. Descriptive Statistics

A total of 61 participants with lateral elbow pain were included in the study. The mean age was 43.6 ± 9.1 years, and 55.70% were male. Most participants were right-handed (91.80%), and just over half were currently employed (54.10%). Detailed Sociodemographic Characteristics of Participants With Lateral Elbow Pain are presented in Table 1.

Regarding the affected side, 60 participants provided valid data. The right elbow was more frequently affected in 36 participants (60%). Pain onset was reported as less than 3 months in 18 participants (30%), 3 months to <6 months in 15 participants (25.0%), the largest proportion 6 months to <2 years in 20 participants (33.30%), and 2 years or more in 7 participants (11.70%). One participant (1.6%) had missing data for this variable. Clinical characteristics of the participant are presented in Table 1.

Participants reported moderate to high pain intensity and variable levels of functional limitation. Median NPRS-Ar and UEFI-Ar scores indicated substantial symptom burden, while PSFS-Ar scores demonstrated a wide range of patient-specific functional limitations. Participants demonstrated variable levels of pain and functional limitation. Descriptive statistics for the PSFS-Ar, NPRS-Ar, and UEFI-Ar are presented in Table 2.

Participants identified activities specifically affected by their condition using the PSFS-Ar, reflecting individualized functional limitations. PSFS-Ar scores ranged from 0.25 to 8.00 (mean = 3.49, SD = 1.94).

Exploratory subgroup comparisons are presented in Table 3. No statistically significant differences were observed according to sex, employment status, or symptom duration. A difference was observed according to the affected side (*p* = 0.03); however, this finding was derived from multiple unadjusted exploratory comparisons and should be interpreted cautiously.

### 3.2. Psychometric Assessment

Neither of the two prespecified construct-validity hypotheses was confirmed. Therefore, construct validity was not supported according to the prespecified hypothesis-testing criterion. The PSFS-Ar demonstrated a weak positive correlation with the UEFI-Ar (r_s_ = 0.35, *p* = 0.005), which was below the prespecified moderate magnitude. The correlation between the PSFS-Ar and NPRS-Ar was negligible and nonsignificant (r_s_ = −0.17, *p* = 0.200), which was also below the prespecified magnitude. Correlation results are presented in Table 4. As a supplementary analysis, Steiger’s Z test indicated that the PSFS-Ar–UEFI-Ar correlation was significantly stronger than the PSFS-Ar–NPRS-Ar correlation (Z = 2.44, *p* = 0.01). This finding supported the predicted relative ordering of the associations but did not establish construct validity. No floor or ceiling effects were observed, as no participants achieved the lowest or highest possible PSFS-Ar scores.

Test–retest reliability was evaluated in a consecutive subsample of 30 participants selected according to their order of recruitment until the planned reliability sample was reached. These participants completed the PSFS-Ar at T1 and T2 over a median interval of 8.5 days (range, 5–14 days). All 30 participants reported no substantial perceived change between assessments and were therefore included in the reliability analysis. However, because no validated stability anchor was used, subtle clinical change could not be excluded. The ICC(2,1) was 0.65 (95% CI: 0.35–0.82), representing a moderate but imprecise point estimate of test–retest reliability.

**Figure 1:**
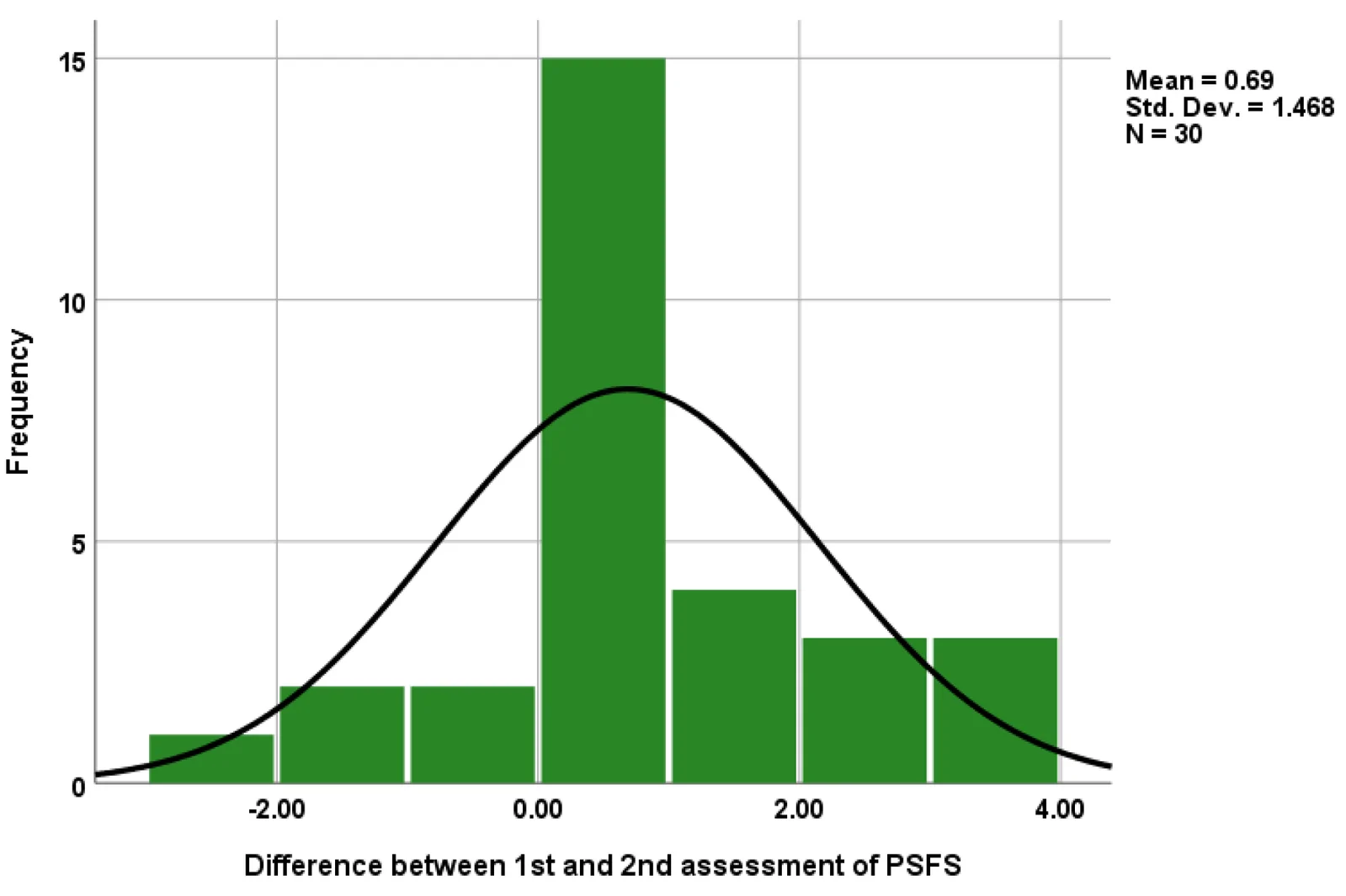
Distribution of Change Scores Between the First and Second PSFS Assessments.

**Figure 2:**
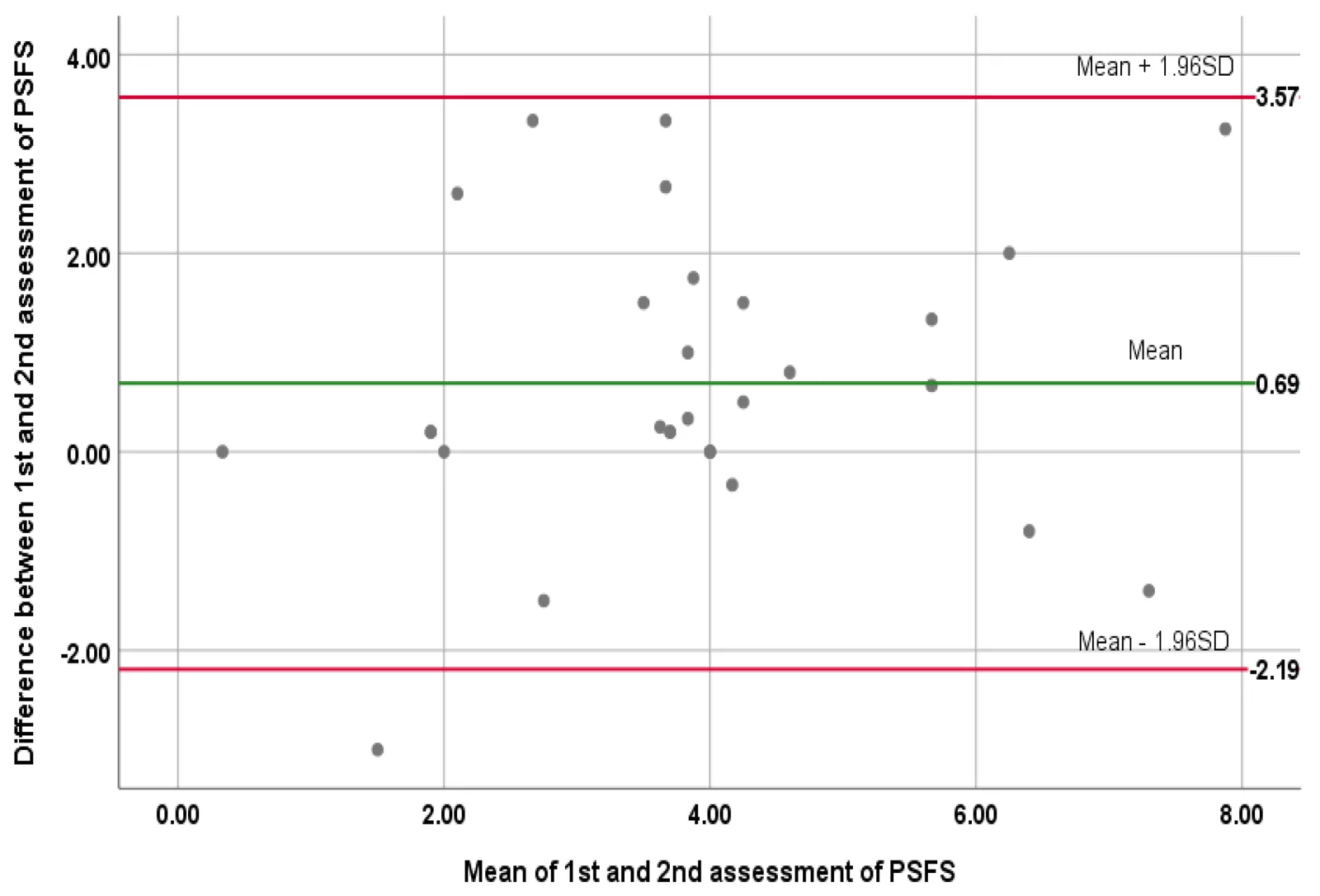
Bland–Altman Plot Assessing Agreement Between the First and Second PSFS Assessments.

The mean PSFS-Ar score was 3.56 ± 1.72 at T1 and 4.24 ± 1.96 at T2. The mean test–retest difference (T2 − T1) was 0.69 points (95% CI: 0.14–1.23), with Bland–Altman limits of agreement ranging from −2.19 to 3.56 points. The confidence interval for the mean difference excluded zero, indicating a systematic increase in scores at retest. Using the baseline SD of 1.72 and ICC(2,1), the SEM was 1.03 points and the MDC95 was 2.86 points. These measurement-error estimates should be interpreted cautiously because clinical stability between assessments could not be confidently established. Reliability results are summarized in Table 5, and agreement plots are shown in Figure 1 and Figure 2.

In the primary reliability analysis involving 30 participants, the ICC(2,1) was 0.65 (95% CI: 0.35–0.82). In the post hoc sensitivity analysis, the ICC was 0.71 (95% CI: 0.42–0.87; *n* = 21) among participants retested within <11 days and 0.74 (95% CI: 0.40–0.90; *n* = 15) among those retested within <9 days. Although the point estimates were higher than in the primary analysis, the confidence intervals remained wide and the analyses involved progressively smaller samples. These exploratory findings should therefore not be interpreted as evidence that shorter retest intervals improve reliability.

## 4. Discussion

The present findings provide preliminary information regarding patient-specific functional status, test–retest reliability, and measurement error. However, responsiveness was not evaluated; therefore, this study does not establish the ability of the PSFS-Ar to detect clinically meaningful change over time. The sample characteristics were broadly consistent with epidemiological data describing LEP as most prevalent in middle-aged adults engaged in repetitive upper-limb loading.

The absence of floor or ceiling effects (<15%) further indicates that the PSFS-Ar can adequately discriminate across a wide spectrum of functional ability, supporting its theoretical suitability for both mild and more severe presentations and mirroring findings from the Arabic PSFS in lower extremity disorders and from PSFS applications in stroke, shoulder, and Dupuytren’s disease [3,6,10].

The PSFS-Ar demonstrated a weak positive correlation with the UEFI-Ar and a negligible, nonsignificant correlation with the NPRS-Ar. These findings suggest that the PSFS-Ar and UEFI-Ar assess related but not identical aspects of upper-extremity function, whereas pain intensity and patient-specific functional performance represent more distinct constructs. Because neither prespecified hypothesis achieved the anticipated magnitude, construct validity was not supported according to the prespecified hypothesis-testing criterion in this sample.

Steiger’s test showed that the PSFS-Ar–UEFI-Ar correlation was significantly stronger than the PSFS-Ar–NPRS-Ar correlation. This finding supports the predicted relative ordering of the associations and is consistent with previous PSFS research showing stronger relationships with functional measures than with pain-intensity measures [3,4,17,26]. Nevertheless, Steiger’s test does not independently establish convergent or discriminant validity, and the modest correlation magnitudes warrant cautious interpretation.

Exploratory subgroup analyses showed no significant differences in PSFS-Ar scores according to gender, work status, or symptom duration. A significant difference was observed between participants with right- and left-elbow involvement. This finding may reflect differences in limb dominance, functional demands, or compensatory strategies; however, because the subgroup analyses were exploratory, not prespecified, and not adjusted for multiple comparisons, these explanations remain tentative. The affected-side finding should therefore be interpreted cautiously and explored further in adequately powered studies designed to examine potential subgroup differences.

The ICC point estimate indicated moderate test–retest reliability (ICC = 0.65). However, the wide 95% confidence interval (0.35–0.82) includes values compatible with poor to good reliability, indicating considerable uncertainty regarding the true magnitude of reliability. In addition, the mean increase of 0.69 points at T2, with a 95% confidence interval excluding zero, suggests that complete clinical stability between assessments cannot be assumed. This systematic increase may reflect spontaneous recovery, treatment exposure, familiarity with the instrument, or other within-person variation during the variable 5–14-day interval. Consequently, the ICC, SEM, and MDC95 estimates should be interpreted cautiously, and the reliability finding should be regarded as a moderate but imprecise point estimate rather than definitive evidence of acceptable reproducibility.

The ICC point estimate was comparable with values reported in the broader PSFS literature, although reliability estimates vary according to clinical condition, study design, retest interval, and participant stability. For example, ICC values of 0.71, 0.87, and 0.95 have been reported in upper-extremity musculoskeletal conditions, primary shoulder complaints, and nonspecific neck pain, respectively [3,10,26,27,28]. Differences between studies may reflect symptom fluctuation, activity exposure, retest timing, and the individualized nature of the PSFS. Small changes in daily task context or symptom interpretation may influence PSFS ratings even when participants report no substantial overall change [3,26].

The higher ICC point estimates observed in the shorter-interval sensitivity analyses may suggest that retest interval contributed to measurement variability. However, the <11-day and <9-day thresholds were post hoc, were not based on established clinical criteria, and produced progressively smaller samples with wide confidence intervals. These analyses therefore provide only exploratory information regarding the robustness of the primary reliability estimate and should not be interpreted as demonstrating an effect of retest interval on reliability.

The SEM was 1.03 points, and the MDC95 was 2.86 points. The MDC95 indicates the magnitude of individual change required to exceed expected measurement error with 95% confidence; it does not indicate whether the change is clinically meaningful or important to the patient.

A low proportion of missing responses (2%) was observed, suggesting good acceptability and usability of the questionnaire. Some participants required clarification regarding the purpose of the questionnaire and how to rate their responses. To ensure accurate completion and minimize missing data, questionnaires were administered in the presence of the research team. This observation highlights the importance of clear instructions when implementing patient-reported outcome measures, as variations in patient familiarity with self-report tools may influence response accuracy and data completeness. Providing brief guidance during administration may therefore enhance the reliability and interpretability of PROM data in clinical research settings [3,6,9].

### Limitations

This study has several limitations that should be considered when interpreting the findings. First, although the sample size met the minimum thresholds used during study planning, the reliability subgroup included only 30 participants. This limited the precision of the ICC, SEM, and MDC95 estimates, as reflected by the wide ICC confidence interval, and may restrict their generalizability. Second, recruitment from a single tertiary hospital in Riyadh may limit generalizability to the wider Arabic-speaking population and other healthcare settings. Future multicenter studies involving more diverse populations are warranted to confirm the reproducibility and external validity of these findings. Third, the test–retest interval also varied from 5 to 14 days, which may have allowed spontaneous symptom fluctuation or recovery to influence the observed scores and affect the reliability and measurement-error estimates. The exact retest interval was not modeled continuously or incorporated as a covariate. The post hoc sensitivity analyses used non-prespecified interval thresholds without established clinical justification. Although included to explore the robustness of the primary estimate to shorter retest intervals, their small sample sizes and wide confidence intervals substantially limit interpretation. Therefore, the ICC, SEM, and MDC95 estimates should be considered preliminary and may not generalize to the wider Arabic-speaking population with lateral elbow pain. Also, the absence of a validated stability anchor means that subtle clinical change may not have been identified, despite participants reporting no substantial change. Lastly, responsiveness was not evaluated because of the cross-sectional design; therefore, the ability of the PSFS-Ar to detect clinically meaningful longitudinal change remains uncertain.

## 5. Conclusions

In Arabic-speaking individuals with lateral elbow pain, the PSFS-Ar demonstrated a moderate but imprecise test–retest reliability estimate. However, the wide ICC confidence interval, small reliability sample, systematic mean change between assessments, variable test–retest interval, and absence of a validated stability anchor limit confidence in the reliability and measurement-error estimates. Neither prespecified construct-validity hypothesis was confirmed; therefore, construct validity was not supported according to the prespecified hypothesis-testing criterion. Accordingly, the present findings should be considered preliminary and do not provide sufficient evidence to establish the reliability or construct validity of the PSFS-Ar specifically in individuals with lateral elbow pain. Larger longitudinal studies using standardized retest, validated stability criteria, and adequately powered samples are required to confirm these measurement properties.

## Figures and Tables

**Table 1:** Sociodemographic and Clinical Characteristics of Participants With Lateral Elbow Pain.

	Min–Max	Mean (SD)	Mode
Age	23–63	43.60 (9.10)	45
		Frequency	Percentage
Gender	Male	34	55.70%
	Female	27	44.30%
Profession	Employed	33	54.10%
	Not employed	16	26.20%
	Retired	9	14.80%
	Unknown	3	4.90%
Dominant hand	Right	56	91.80%
	Left	5	8.20%
* Affected side (valid *n* = 60)	Right elbow	36	60.00
	Left elbow	24	40.00
* Pain onset (valid *n* = 60)	<3 months	18	30.00
	3 to <6 months	15	25.00
	6 months to <2 years	20	33.30
	≥2 years	7	11.70

* Percentages for affected side and pain onset are based on available data (valid *n* = 60 for each variable). One observation was missing for affected side and one for pain onset.

**Table 2:** Descriptive Statistics for Pain and Functional Outcome Measures.

Scale	N	Min	Max	Descriptive Statistic
PSFS-Ar	61	0.25	8.00	Mean (SD): 3.49 (1.94)
LEP NPRS-Ar	61	2	10	Median (IQR): 7.00 (3.00)
UEFI-Ar	61	4	73	Median (IQR): 43.00 (26.00)

PSFS-Ar: Arabic version of Patient-Specific Functional Scale; LEP NPRS: Lateral Elbow Pain of the Arabic Version of the Numeric Pain Rating Scale; UEFI-Ar: Arabic Version of Upper Extremity Functional Index; Normally distributed variables are presented as mean and Standard Deviation (SD); non-normally distributed variables are presented as median and Interquartile range (IQR).

**Table 3:** Comparison of PSFS-Ar Scores Across Participant Demographic and Clinical Characteristics.

		PSFS Score					Test Statistic	*p*-Value
	(Valid Responses)	M	SD	Median	IQR	Test		
Gender	Male (34)	3.69	1.97	3.90	2.81	Mann–Whitney U	Z = −1.23	0.22
	Female (27)	3.24	1.90	2.75	2.00			
Work	Not working (16)	2.88	1.86	2.00	2.13	Kruskal–Wallis H	H = 3.38	0.19
	Working (33)	3.69	2.06	3.60	3.12			
	Retired (9)	3.84	1.42	4.33	2.46			
Affected elbow	Left (24)	2.85	1.87	2.33	1.89	Independent-samples *t* test	T = 2.24	0.03
	Right (36)	3.96	1.88	4.00	2.88			
Onset of pain	<3 months (18)	3.14	1.83	3.00	2.30	One-way ANOVA	F = 0.26	0.85
	3 to <6 months (15)	3.34	2.19	3.50	4.33			
	6 months to <2 years (20)	3.65	1.71	3.58	3.00			
	≥2 years (7)	3.60	1.88	3.60	2.00			

SD, standard deviation; IQR, interquartile range. Statistical tests are identified directly in the table. These analyses were exploratory and were not adjusted for multiple comparisons; therefore, findings should not be interpreted as confirmatory.

**Table 4:** Spearman’s Correlations Between PSFS-Ar, LEP NPRS-Ar, and UEFI-Ar.

Variable	PSFS-Ar	*p*-Value	LEP NPRS-Ar	*p*-Value	UEFI-Ar
PSFS-Ar	1.00				
LEP NPRS-Ar	−0.17 ^a^	0.2	1.000		
UEFI-Ar	0.35 ^a^	0.005	−0.41 ^a^	0.001	1.00
N	61		61		61

^a^ Spearman’s rho; LEP NPRS-Ar: Lateral Elbow Pain of the Arabic Version of the Numeric Pain Rating Scale; UEFI-Ar: Arabic Version of Upper Extremity Functional Index; PSFS-Ar: Arabic version of Patient-Specific Functional Scale.

**Table 5:** Test–Retest Reliability of the PSFS-Ar in the Reliability Subsample (*n* = 30) Using ICC(2,1).

ICC Type	ICC (95% CI)	F (df1, df2)	*p*-Value
Single Measures	0.65 (0.35–0.82)	5.30 (29, 29)	<0.001

ICC: Intraclass Correlation Coefficient.

## Data Availability

The data are not publicly available due to ethical and privacy restrictions. However, they are available from the corresponding author upon reasonable request and with appropriate ethical approval.

## Abbreviations

The following abbreviations are used in this manuscript:

COSMIN	Consensus-based Standards for the Selection of Health Measurement Instruments
CPGs	Clinical Practice Guidelines
ICC	Intraclass Correlation Coefficient
IQR	Interquartile Range
LEP	Lateral Elbow Pain
MCID	Minimal Clinically Important Difference
MDC95	Minimal Detectable Change at 95% Confidence Level
NPRS	Numeric Pain Rating Scale
NPRS-Ar	Arabic Version of the Numeric Pain Rating Scale
PROMs	Patient-Reported Outcome Measures
PSFS	Patient-Specific Functional Scale
PSFS-Ar	Arabic Version of the Patient-Specific Functional Scale
SD	Standard Deviation
SEM	Standard Error of Measurement
UEFI	Upper Extremity Functional Index
UEFI-Ar	Arabic Version of the Upper Extremity Functional Index
